# The mitochondrial genome of the egg-laying flatworm *Aglaiogyrodactylus forficulatus* (Platyhelminthes: Monogenoidea)

**DOI:** 10.1186/s13071-016-1586-2

**Published:** 2016-05-17

**Authors:** Lutz Bachmann, Bastian Fromm, Luciana Patella de Azambuja, Walter A. Boeger

**Affiliations:** Natural History Museum, University of Oslo, PO Box 1172, Blindern, 0318 Oslo Norway; Department of Tumor Biology, Institute for Cancer Research, Norwegian Radium, Hospital, Oslo University Hospital, PO Box 4950, Nydalen, 0424 Oslo Norway; Laboratório de Ecologia Molecular e Parasitologia Evolutiva-LEMPE, Universidade, Federal do Paraná-UFPR, Curitiba, Brazil

**Keywords:** Brazil, Fish parasites, Gene order, Gyrodactylidae, Mitogenomics, Monogenoidea, Neodermata

## Abstract

**Background:**

The rather species-poor oviparous gyrodactylids are restricted to South America. It was suggested that they have a basal position within the otherwise viviparous Gyrodactylidae. Accordingly, it was proposed that the species-rich viviparous gyrodactylids diversified and dispersed from there.

**Methods:**

The mitochondrial genome of *Aglaiogyrodactylus forficulatus* was bioinformatically assembled from next-generation illumina MiSeq sequencing reads, annotated, and compared to previously published mitochondrial genomes of other monogenoidean flatworm species.

**Results:**

The mitochondrial genome of *A. forficulatus* consists of 14,371 bp with an average A + T content of 75.12 %. All expected 12 protein coding, 22 tRNA, and 2 rRNA genes were identified. Furthermore, there were two repetitive non-coding regions essentially consisting of 88 bp and 233 bp repeats, respectively. Maximum Likelihood analyses placed the mitochondrial genome of *A. forficulatus* in a well-supported clade together with the viviparous Gyrodactylidae species. The gene order differs in comparison to that of other monogenoidean species, with rearrangements mainly affecting tRNA genes. In comparison to *Paragyrodactylus variegatus*, four gene order rearrangements, i.e. three transpositions and one complex tandem-duplication-random-loss event, were detected.

**Conclusion:**

Mitochondrial genome sequence analyses support a basal position of the oviparous *A. forficulatus* within Gyrodactylidae, and a sister group relationship of the oviparous and viviparous forms.

**Electronic supplementary material:**

The online version of this article (doi:10.1186/s13071-016-1586-2) contains supplementary material, which is available to authorized users.

## Background

Within flatworms (Platyhelminthes) the parasitic Neodermata represent the most derived forms and are, at least when compared to the free-living flatworm lineages, particularly species rich. They include the ectoparasitic Monogenoidea, and the endoparasitic flukes (Trematoda) and tapeworms (Cestoda).

Among the Monogenoidea, Gyrodactylidae have attracted particular attention as some species have been of great concern for humans. For example, *Gyrodactylus salaris* Malmberg, 1957 has caused significant ecological and economic damage to wild stocks of Atlantic salmon (*Salmo salar*) in Norway and Russia as well as in aquaculture [1]. The majority of the species within Gyrodactylidae are hyperviviparous ectoparasites on actinopterygian fish hosts, but there are also some oviparous gyrodactylid lineages. These lineages were originally placed within the Oogyrodactylidae [2], which was later rejected as paraphyletic by Boeger et al. [3], and Oogyrodactylidae were included in Gyrodactylidae based on morphological synapomorphies.

Oviparous gyrodactylids are restricted to South America and occur on freshwater catfishes, mainly on species of the Loricariidae (Siluriformes). Boeger et al. [4] considered the oviparous species a basal lineage within Gyrodactylidae. Taking into account their geographical restriction to continental South America they argued that the species-rich viviparous gyrodactylids diversified and dispersed from there [4]. Accordingly, hyperviviparity and the loss of ‘sticky’ eggs were interpreted as synapomorphic key innovations of the viviparous Gyrodactylidae.

In comparison to the enormous diversity of the viviparous gyrodactylids the oviparous lineages appear rather species-poor; until today only 23 species in seven genera have been described [5]. Among them is *Aglaiogyrodactylus forficulatus* Kritsky, Vianna & Boeger, 2007, the type-species of the genus, that was originally described from the loricarid host *Kronichthys lacerta* [5].

In the current study we present the first mitochondrial genome of an oviparous gyrodactylid. The molecular characteristics of the mitochondrial genome of *A. forficulatus* are compared with those of other previously described monogenoidean mitochondrial genomes, and the phylogenetic position of oviparous gyrodactylids is addressed.

## Methods

The oviparous *A. forficulatus* was collected from its type-host, the catfish *Kronichthys lacerta* Nichols, 1919*,* from Rio Morato, basin of the Garaqueçaba, Paraná, Brazil (25°12'48''S, 48°17'52''W) on 30 October 2013.

About 200 individual parasites were pooled for DNA extraction with the E.Z.N.A. Tissue DNA kit (Omega Bio-Tek) following the Tissue DNA-Spin Protocol provided with the kit. For high-throughput next-generation sequencing (NGS) of the genomic DNA paired-end libraries were prepared, tagged, and analyzed (29,107,020 paired 300 bp reads) on an illumina MiSeq (outsourced to GENterprise GENOMICS, Mainz, Germany).

The mitochondrial genome of *A. forficulatus* was reconstructed by assembling the NGS reads using MITObim 1.8 [6] using essentially the default settings of the program, and, in addition, the program’s quality trimming option. The COII sequence of *A. ctenistus* (GenBank accession number KF751723; [7]) was used as seed sequence for the assembly.

Annotation of the mitochondrial genes was done using MITOS [8] and DOGMA [9]. In addition, tRNA genes were also identified using tRNAscan-SE 1.21 [10]. For most protein coding genes (PCGs) only the conserved domains were identified by the two annotation programs. Phylogenetic comparisons with already published monogenoidean mitochondrial genomes (Table 1) were, therefore, used to manually complete these genes’ annotation, which was assisted by blast searches of GenBank. Codon usage was analyzed using the Sequence Manipulation Suite v2 [11]. The dot-plot approach of YASS [12] was used to identify and visualize the repeat regions. The mitochondrial gene map was drawn using SnapGene 3.0.

**Table 1:** The mitochondrial genomes of 11 monogenoidean and two cestode species included in this study for comparative analyses

Species	GenBank accession number	Length (bp)	References
Monogenoidea: Gyrodactylidea			
* Aglaiogyrodactylus forficulatus*	KU679421	14,371	this study
* Paragyrodactylus variegatus*	KM067269	14,517	[13]
* Gyrodactylus salaris* ^*a*^	NC008815	14,790	[14]
* Gyrodactylus thymalli* ^*a*^	NC009682	14,788	[15]
* Gyrodactylus derjavinoides*	NC010976	14,741	[16]
Monogenoidea: Capsalidea			
* Benedenia seriolae*	HM222526	13,498	[17]
* Benedenia hoshinai*	EF055880	13,554	[18]
* Neobenedenia melleni*	JQ038228	13,270	[19]
Monogenoidea: Dactylogyridea			
* Tetrancistrum nebulosi*	NC018031	13,392	[20]
Monogenoidea: Mazocraeidea			
* Polylabris halichoeres*	NC016057	15,527	[21]
* Microcotyle sebastis*	NC009055	14,407	[22]
* Pseudochauhanea macrorchis*	NC016950	15,031	[23]
Cestoda			
* Hymenolepis diminuta*	AF314223	13,900	[24]
* Echinococcus oligarthrus*	NC009461	13,791	[25]

^a^recently synonymized by [35]

The assembled mitochondrial genome of *A. forficulatus* was compared to previously published mitochondrial genomes of other monogenoidean species [13–25] that are listed in Table 1. The mitochondrial genomes of the cestodes *Echinococcus oligarthrus* [24] and *Hymenolepis diminuta* [25] served as outgroups. All genes were aligned individually using the online version of MAFFT Alignment v7.245 [26]. Subsequently the individual tRNA, rRNA, and PCG alignments were concatenated into one extended alignment, which did not include the non-coding regions of the mitochondrial genes. GBlock v0.91b [27] and GUIDANCE2 [28] were applied to remove ambiguous and unreliable sections from the concatenated MAFFT alignments.

PartitionFinder v.1.1.1_Mac [29] was used to select the best-fit model of molecular evolution for the concatenated alignments using as recommended the program’s “raxml” search option and the Bayesian Information Criterion (BIC) for model selection. Maximum Likelihood analyses were carried out in PHYML 3.0 [30] applying the GTR + I + G model and 1,000 bootstrap replicates for all analyses except those for the Cytb and ND3 genes that were performed applying the GTR + G model.

Comparisons of gene orders of the mitochondrial genomes of *A. forficulatus* and the other monogenoidean species included in this study were conducted in CREx [31], a program that infers most parsimonious gene rearrangements based on common intervals.

## Ethics approval

All sampling of parasites (on fish) was done under license number 10007 (Instituto Chico Mendes de Conservação da Biodiversidade - ICMBio, Brazil).

## Results and discussion

Next-generation sequencing of the genomic DNA of *A. forficulatus* delivered 29,107,020 million paired 300 bp reads. The initial assembly with the COII sequence of *A. ctenistus* as seed sequence as well as subsequent control assemblies using various *A. forficulatus* seeds delivered identical mitochondrial DNA (mtDNA) consensus sequences. During the assembly of the mtDNA of *A. forficulatus* particular attention was paid to the two repetitive repeat regions (see below) in order to avoid erroneous base-calling due to misassembled short reads; the final sequence was determined only from those reads that also in part covered the respective flanking regions.

The mtDNA of *A. forficulatus* is 14,371 bp long, and was assembled from 43,334 quality-trimmed reads with an average coverage of 765 x. The overall A + T content was 75.1 % and the nucleotide composition was A (30.2 %) C (9.7 %), G (15.1 %), and T (44.9 %). The base composition of the tRNA and rRNA genes, PCGs, and repeat regions are listed in Additional file 1. The nucleotide sequence of the assembled mtDNA of *A. forficulatus* was deposited in GenBank with the accession number KU679421.

**Fig. 1:**
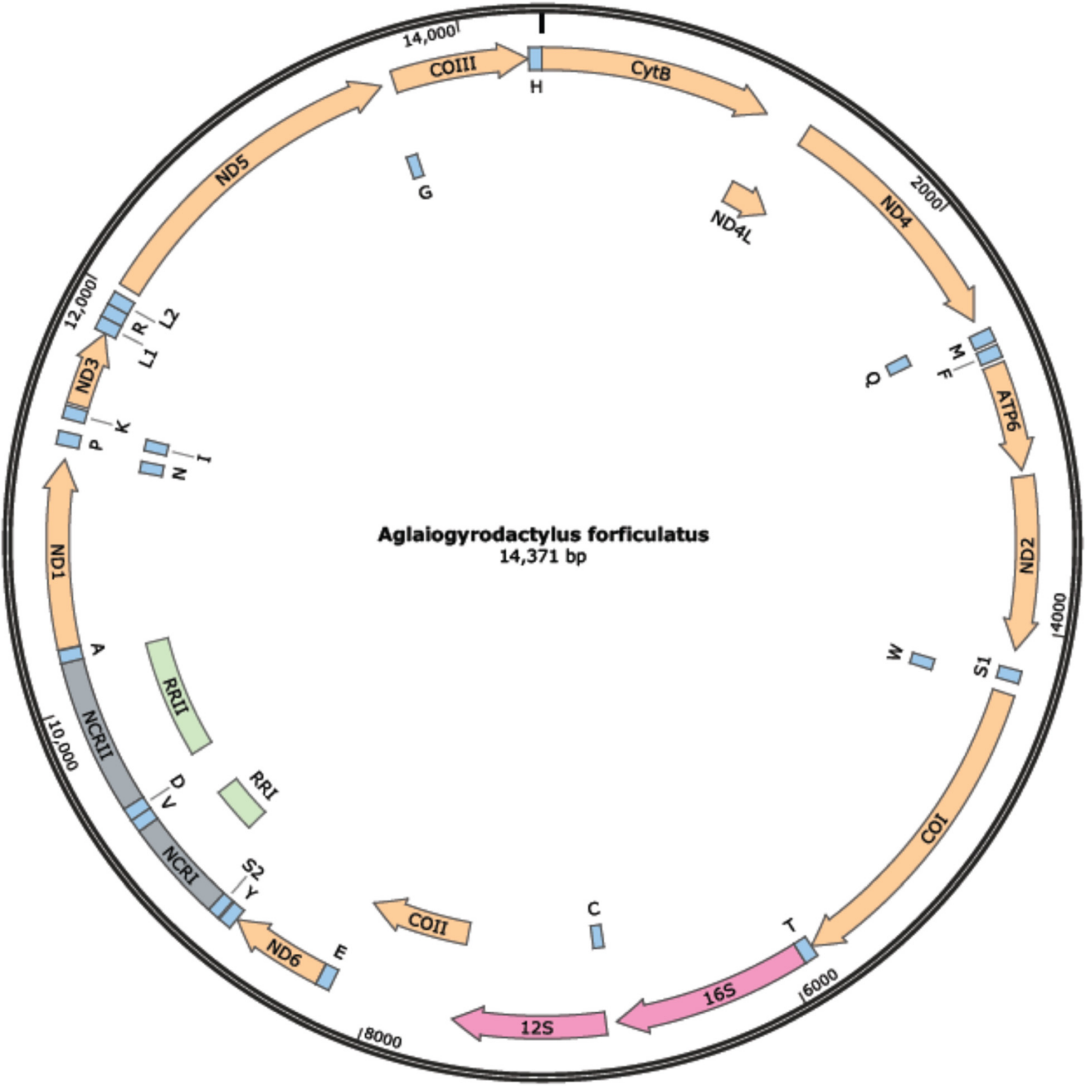
Map of the mitochondrial genome of *Aglaiogyrodactylus forficulatus*. The 12 protein coding, 22 tRNA, and two rRNA genes are depicted as well as the non-coding regions (NCR) I and II and the respective repeat regions (RR) I and II

All genes usually described for the mitochondrial genomes of other Monogenoidea (12 protein coding genes, two rRNAs, and 22 tRNAs) were also identified for *A. forficulatus* (Fig. 1, Table 2). Accordingly, ATP8 lacks also in *A. forficulatus*. All genes are coded on the same strand.

**Table 2:** The mitochondrial genome of *Aglaiogyrodactylus forficulatus*; gene organization and gene order as determined by MITOS [8] and DOGMA [9]

Gene	Position	Length (bp)	Start/Stop codon of PCGs	Best-fit model^c^
Cytb	1–1101	1101	ATG/TAG	GTR + G
ND4L	1109–1363	255	ATG/TAA	GTR + I + G
ND4	1306–2508	1203	ATT/TAG	
tRNA Q	2504–2566	63		GTR + I + G
tRNA M^a^	2568–2632	65		GTR + I + G
tRNA F^a^	2651–2713	63		GTR + I + G
ATP6	2735–3250	516	ATG/TAA	GTR + I + G
ND2	3280–4104	825	ATT/TAG	GTR + I + G
tRNA S1	4193–4251	59		GTR + I + G
tRNA W	4252–4314	63		GTR + I + G
COI	4312–5841	1530	ATG/TAG	GTR + I + G
tRNA T^a^	5842–5906	65		GTR + I + G
16S	5907–6834	928		GTR + I + G
tRNA C	6843–6901	59		GTR + I + G
12S	6887–7615	729		GTR + I + G
COII	7613–8188	576	ATG/TAG	GTR + I + G
tRNA E	8205–8270	66		GTR + I + G
ND6	8272–8742	471	ATG/TAA	GTR + I + G
tRNA Y	8746–8808	63		GTR + I + G
tRNA S2	8817–8873	57		GTR + I + G
Non–coding region I	8874–9358	485		
Repeat region I	9017–9277	261		
tRNA V	9359–9420	62		GTR + I + G
tRNA D^a^	9421–9482	62		GTR + I + G
Non–coding region II	9483–10216	733		
Repeat region II	9539–10212	675		
tRNA A^a^	10217–10277	61		GTR + I + G
ND1	10282–11175	894	ATG/TAG	
tRNA N^a^	11175–11237	63		GTR + I + G
tRNA P^b^	11238–11301	64		GTR + I + G
tRNA I	11300–11362	63		GTR + I + G
tRNA K^a^	11363–11423	61		GTR + I + G
ND3	11438–11800	363	ATG/TAG	GTR + G
tRNA L1^a^	11801–11865	65		GTR + I + G
tRNA R	11866–11931	66		GTR + I + G
tRNA L2	11932–11995	64		GTR + I + G
ND5	12038–13597	1560	ATG/TAA	GTR + I + G
tRNA G	13597–13658	62		GTR + I + G
COIII	13659–14304	645	ATG/TAA	GTR + I + G
tRNA H	14308–14369	63		GTR + I + G

^a^genes confirmed by tRNAscan-SE [10]; ^b^tRNA P was only identified by MITOS [8]; ^c^using PartitionFinder v.1.1.1_Mac [29] with the program’s “raxml” search option and the Bayesian Information Criterion (BIC), all tRNAs were concatenated into one partition

### Protein coding genes (PCGs)

For most PCGs the applied annotation programs only detected the conserved domains. This worked best for the COI and Cytb genes, whereas ND2 was not detected at all. Thus, most PCGs were annotated manually through phylogenetic comparisons with reference to other previously published platyhelminth mitochondrial genomes (Table 1). Translation into amino acid sequences was straightforward using the flatworm mitochondrial code [32]. All PCG except for ND2 and ND4 use ATG as start codon. For ND2 and ND4, however, it proved difficult to identify the beginning of the gene, as there was no canonical start codon of flatworm mitochondria (reviewed in [33]) in frame in the respective region. As a working hypothesis, we suggest that both ND2 and ND4 start with ATT, which was reported earlier as a rarely used alternative start codon in other flatworm mitochondria (e.g. [13, 34]). All PCGs terminate with one of the canonical flatworm stop codons TAG or TAA (Table 2). As reported for other monogenoidean mitochondrial genomes, *A. forficulatus* shows a strong codon bias for PCGs. The most commonly used codons for a particular amino acid in *A. forficulatus* were, however, in all cases the same as for *P. variegatus* (Additional file 2).

### rRNA genes

The 12S and 16S rRNA genes were identified of being 729 bp and 928 bp long, respectively. The A + T – content was 77.4 % for the 12S gene and for the 75.6 % 16S rRNA gene, and thus very similar to the average of the whole mitochondrial genome.

### tRNA genes

All 22 tRNA genes were identified by MITOS [8] and DOGMA [9] that, however, failed to detect tRNA P for prolin. tRNAscan-SE [10] confirmed eight tRNAs but could not predict the remaining ones (Table 2). The secondary cloverleaf structures of the predicted tRNAs are compiled in Additional file 3. The tRNAs C, S1, and S2 lack the DHU arm.

## Genetic diversity and phylogenetic analyses

**Fig. 2:**
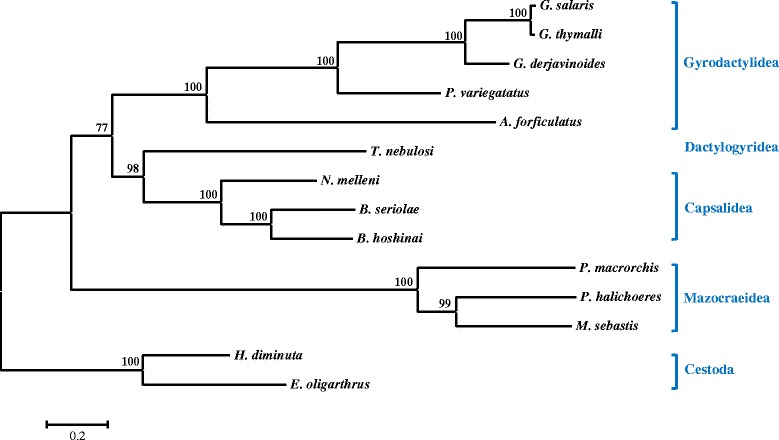
Maximum Likelihood tree based on the concatenated MAFFT alignments of mitochondrial genes of *A. forficulatus* and 11 further Monogenoidea species. The cestode species *H. diminuta* and *E. oligarthrus* served as outgroup. Bootstrap support values are indicated

Based on the concatenated MAFFT alignments (15,261 positions), the mitochondrial genome of the egg laying *A. forficulatus* was found in a sister-group relationship to the mitochondrial genomes of the other viviparous Gyrodactylidea, i.e. *G. salaris*, *G. thymalli*, *G. derjavinoides*, and *P. variegatus* (Fig. 2) with high statistical support. However, it has to be taken into account that the depicted ML tree does not provide a comprehensive phylogenetic hypothesis for the Monogenoidea since it is only based on the limited number of available mitochondrial genomes. All included Capsalidea, Mazocraeidea, respectively, also clustered together with high statistical support. Despite the limitations of the analyses the observed phylogenetic affinity of *A. forficulatus* to the included viviparous Gyrodactylidea species makes sense, as the grouping is congruent with the results of earlier morphological analyses [3].

Similar tree topologies with respect to the Gyrodactylidea clade were also obtained when analyzing the concatenated tRNA (bootstrap support 50) genes as well as the COI (100), COII (86), COIII (65), Cytb (77), ND1 (100), ND3 (66), ND4L (93), ND4 (99), and ND5 (100) genes individually. When analyzing the rRNA genes as well as the ATP6, ND2, and ND6 genes individually somewhat different tree topologies with affinities of *A. forficulatus* to other species or clades were obtained. Similar tree topologies were also obtained when using the concatenated GBlock and GUIDANCE2 alignments, respectively (Additional file 4). With the unreliable parts of the original MAFFT alignment removed, the clade consisting of species of Gyrodactylidae received a bootstrap support of 100.

The clustering of the mitochondrial genome of *A. forficulatus* with the remaining Gyrodactylidae species is nevertheless not strongly reflected in a matrix of pairwise genetic distances. Although the genetic distance between *A. forficulatus* and the closest relative *P. variegatus* was lowest (0.402; p-distances), the obtained values for all other pairwise comparisons were only slightly higher ranging from 0.409 with *N. melleni* to 0.477 with *M. sebastis* (Additional file 5). The respective values for the GBlock alignment were 0.396 for the comparison with *P. variegatus* and a range from 0.402 with *N. melleni* to 0.471 with *M. sebastis* (Additional file 5).

### Non-coding regions (NCRs) including repeat regions I and II

Two extended non-coding regions of 486 bp and 733 bp, respectively, were detected in the mitochondrial genome of *A. forficulatus*. They are located relatively close to each other, separated only by the tRNA V and tRNA D genes, i.e. 123 bp, within a cluster of five tRNA genes that represents in comparison to other previously published monogenoidean mitochondrial genomes a substantially rearranged part of the mtDNA (Additional file 6). Both non-coding regions include a repetitive region consisting of three repeats. In repeat region I the repeats are 88 bp long while they span 233 bp in repeat region II. For both repetitive sequence motifs blast searches revealed no significant sequence similarity to other GenBank entries. The average sequence similarity between the repeats was 81.1 % in repeat region I and 96.5 % in repeat region II, with somewhat higher sequence divergence in the beginning and the end of the repeated arrays (Additional file 7). The AT-content of repeat region I is 75.48 %, and repeat region II is particularly AT rich (83.38 %) and may contain important functional domains for replication and transcription. However, such functional domains have not been described yet in monogenoidean flatworms in more detail than e.g. “Poly T stretch” or “A + T – rich segment” [13], and similar domains can be certainly found in the repeat region of *A. forficulatus* as well. Nevertheless, without a comprehensive functional analysis the identification of such functional domains would remain highly speculative.

The structure of the non-coding regions of *A. forficulatus* is strikingly similar to that of *P. variegatus* [13] although there is only one non-coding region in the latter species. The non-coding region in *P. variegatus* also consists essentially of two repetitive regions, one consisting of two 394 bp repeats (termed part I and II), and one consisting of three 81 bp repeats (termed part III). In contrast, the two non-coding regions observed in the mitochondrial genomes of the other included Gyroadactylidae species *G. salaris* [14], *G. thymalli* [15], and *G. derjavinoides* [16] do not consist of internal repeats. However, in these species the sequences of the two repeat regions are highly similar to each other indicating on the one hand that they originated from a duplication and on the other hand that they bear some functional domains.

## Gene order

**Fig. 3:**
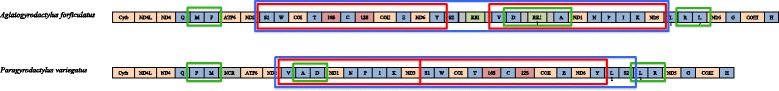
Recombination scenario proposed by CREx [31] to explain mitochondrial gene order changes between *A. forficulatus* and *P. variegatus*. This includes three transpositions (green boxes) and one complex tandem-duplication-random-loss (tdrl) event (red and blue boxes)

As proposed by the CREx program [30] *P. variegatus* and *T. nebulosi* (both species share identical mitochondrial gene orders except for the repetitive non-coding region in *P. variegatus*) have the highest similarity values in gene order based on inferred common intervals (Additional file 8). The program suggested a recombination scenario consisting of three transpositions and one complex tandem-duplication-random-loss (tdrl) event. The proposed transpositions (inversions; green boxes in Fig. 3) affect (a) tRNA-F and tRNA-M, (b) tRNA-A and tRNA-D, and (c) tRNA-R and tRNA-L2, and the proposed tdrl affect two larger gene blocks (red and blue boxes in Fig. 3). However, the proposed tdrl event can alternatively also be interpreted as a series of transpositions. Four larger gene blocks are conserved over all Gyrodactylidae species included in this comparison (Additional file 6). Rearrangements of gene order have already been described for several mitochondrial genomes of Neodermata species (e.g. [13]). The four rearrangements in gene order of *A. forficulatus* in comparison to *P. variegatus* are thus not very surprising. As reported for other Monogenoidea mitochondrial gene order rearrangements mainly affect the specific position of some tRNA genes and non-coding regions. Also in *A. forficulatus* individual protein coding genes are not rearranged. For the mitochondrial genomes of the Gyrodactylidea species included in this study there are essentially four conserved regions; these are (i) ND5, tRNA G, COIII, tRNA H, Cytb, ND4L, ND4, (ii) ATP6, ND2, (iii) tRNA S1, tRNA W, COI, tRNA T, 16S, tRNA C, 12S, COII, tRNA E, ND6, tRNA Y, and (iv) ND1, tRNA N, tRNA P, tRNA I, tRNA K, ND3 (Additional file 6).

## Conclusions

The mitochondrial genome of *A. forficulatus* shows a hitherto unique gene order within the monogenoiden Gyrodactylidea with four rearrangements in comparison to *P. variegatus*. The previously proposed sister group relationship of the oviparous and viviparous Gyrodactylidae is corroborated. However, more comprehensive sampling is required to further test the proposed phylogenetic hypothesis. All Gyrodactylidea mitochondrial genomes sequenced so far include repetitive regions, although the structure of two regions consisting of short tandemly arranged repeats that was found in the basal Gyrodactylidea lineages represented by *A. forficulatus* and *P. variegatus* differs substantially from the structure of two longer dispersed repeats in *Gyrodactylus* spp. The biological function of these repetitive regions is yet unknown but the sequencing of mitochondrial genomes of further Gyrodactylidae species may shed some light on the evolution of these regions.

## Additional files

Additional file 1: Table S1. Base composition of the mtDNA. (DOCX 53 kb) Additional file 2: Table S2. Codon usage of the protein coding genes (PCGs). (DOCX 107 kb) Additional file 3: Figure S1. The secondary structures of the tRNAs. (PDF 184 kb) Additional file 4: Figure S2. Maximum Likelihood trees after removal of ambiguous sites. (PDF 119 kb) Additional file 5: Table S3. P-distances of concatenated mitochondrial genes. (DOCX 17 kb) Additional file 6: Figure S3. Schematic order of the mitochondrial genes. (PDF 113 kb) Additional file 7: Figure S4. Nucleotide sequence alignment of the repeat regions I and II. (DOCX 82 kb) Additional file 8: Table S4. CREx distance matrix for mitochondrial gene order rearrangements. (DOCX 16 kb)
