# Supplementary material for: The mitochondrial genome of the egg-laying flatworm Aglaiogyrodactylus forficulatus (Platyhelminthes: Monogenoidea)
Source: Parasit Vectors. 2016 May 17;9:285. doi: 10.1186/s13071-016-1586-2 (PMC4869361; doi:10.1186/s13071-016-1586-2)
Supplement: Additional file 1: Table S1. — Base composition of the mtDNA. (DOCX 53 kb) [file 13071_2016_1586_MOESM1_ESM.docx]

**Additional File 1:** Base composition, A+T and C+G content, as well as A/T and C/G ratios of the complete mitochondrial genome of *Agaliogyrodactylus forficulatus*, and the rRNA, tRNA, and protein-coding (PCG) genes, as well as the non-coding regions (NCR). All values relate to the coding strand and are given in % except for the A/T and C/G ratios.

|  | A | C | G | T | A+T | C+G | A/T | C/G |
| --- | --- | --- | --- | --- | --- | --- | --- | --- |
| complete | 30.2 | 9.7 | 15.1 | 44.9 | 75.1 | 24.8 | 0.67 | 0.64 |
| PCGs | 27.8 | 10.4 | 15.5 | 46.3 | 74.1 | 25.9 | 0.59 | 0.67 |
| rRNAs | 36.7 | 8.9 | 14.5 | 39.8 | 76.5 | 23.4 | 0.92 | 0.61 |
| tRNAs | 34.5 | 9.9 | 15.9 | 39.8 | 74.3 | 25.8 | 0.87 | 0.62 |
| NCR I | 36.6 | 11.7 | 11.9 | 39.7 | 76.3 | 23.6 | 0.92 | 0.98 |
| NCR II | 34.4 | 6.6 | 10.4 | 48.7 | 83.1 | 17.0 | 0.71 | 0.63 |
