# Supplementary material for: The mitochondrial genome of the egg-laying flatworm Aglaiogyrodactylus forficulatus (Platyhelminthes: Monogenoidea)
Source: Parasit Vectors. 2016 May 17;9:285. doi: 10.1186/s13071-016-1586-2 (PMC4869361; doi:10.1186/s13071-016-1586-2)
Supplement: Additional file 2: Table S2. — Codon usage of the protein coding genes (PCGs). (DOCX 107 kb) [file 13071_2016_1586_MOESM2_ESM.docx]

**Additional File 2:** Codon usage of the protein coding genes (PCGs) of the mitochondrial genome of *A. forficulatus*. The most commonly used codons are highlighted.

|  | **Cytb** | **ND4L** | **ND4** | **ATP6** | **ND2** | **COI** | **COII** | **ND6** | **ND1** | **ND3** | **ND5** | **COIII** | **Sum** | **% usage** |
| --- | --- | --- | --- | --- | --- | --- | --- | --- | --- | --- | --- | --- | --- | --- |
| **GCG (Ala)** | 2 | 0 | 3 | 1 | 0 | 1 | 0 | 0 | 1 | 0 | 0 | 1 | 9 | 9.09 |
| **GCA (Ala)** | 1 | 2 | 1 | 2 | 0 | 6 | 0 | 4 | 2 | 0 | 5 | 0 | 23 | 23.23 |
| **GCT (Ala)** | 11 | 2 | 8 | 4 | 2 | 12 | 1 | 1 | 2 | 2 | 9 | 4 | 58 | **58.59** |
| **GCC (Ala)** | 1 | 0 | 3 | 0 | 0 | 1 | 0 | 0 | 2 | 0 | 2 | 0 | 9 | 9.09 |
| **TGT (Cys)** | 5 | 0 | 5 | 1 | 5 | 8 | 7 | 7 | 11 | 2 | 9 | 2 | 62 | **88.57** |
| **TGC (Cys)** | 1 | 1 | 2 | 0 | 0 | 0 | 1 | 0 | 1 | 1 | 1 | 0 | 8 | 11.43 |
| **GAT (Asp)** | 6 | 0 | 2 | 1 | 1 | 9 | 5 | 0 | 3 | 2 | 6 | 5 | 40 | **75.47** |
| **GAC (Asp)** | 2 | 0 | 2 | 0 | 0 | 1 | 3 | 0 | 0 | 2 | 1 | 2 | 13 | 24.53 |
| **GAG (Glu)** | 4 | 1 | 3 | 1 | 1 | 1 | 2 | 0 | 3 | 1 | 2 | 1 | 20 | 32.79 |
| **GAA (Glu)** | 1 | 1 | 5 | 2 | 2 | 5 | 5 | 3 | 6 | 2 | 5 | 4 | 41 | **67.21** |
| **TTT (Phe)** | 29 | 9 | 43 | 26 | 49 | 51 | 9 | 20 | 42 | 14 | 67 | 25 | 384 | **95.52** |
| **TTC (Phe)** | 3 | 0 | 4 | 2 | 1 | 3 | 0 | 0 | 1 | 1 | 1 | 2 | 18 | 4.48 |
| **GGG (Gly)** | 7 | 0 | 7 | 2 | 2 | 10 | 2 | 1 | 4 | 2 | 3 | 0 | 40 | 21.28 |
| **GGA (Gly)** | 4 | 2 | 6 | 2 | 2 | 15 | 3 | 5 | 4 | 0 | 13 | 6 | 60 | 31.91 |
| **GGT (Gly)** | 9 | 0 | 3 | 3 | 2 | 12 | 6 | 5 | 10 | 4 | 9 | 5 | 68 | **36.17** |
| **GGC(Gly)** | 5 | 0 | 2 | 1 | 0 | 5 | 0 | 0 | 2 | 1 | 3 | 1 | 20 | 10.64 |
| **CAT (His)** | 9 | 0 | 4 | 1 | 0 | 10 | 2 | 1 | 3 | 0 | 6 | 7 | 43 | **78.18** |
| **CAC (His)** | 2 | 0 | 1 | 2 | 0 | 4 | 2 | 0 | 0 | 0 | 1 | 0 | 12 | 21.82 |
| **ATA (Ile)** | 19 | 10 | 27 | 9 | 12 | 24 | 7 | 7 | 11 | 7 | 27 | 8 | 168 | 42.21 |
| **ATT (Ile)**** | 16 | 11 | 35 | 10 | 17 | 25 | 12 | 11 | 19 | 8 | 38 | 10 | 212 | **53.27** |
| **ATC (Ile)** | 1 | 1 | 2 | 1 | 1 | 4 | 1 | 1 | 2 | 0 | 3 | 1 | 18 | 4.52 |
| **AAG (Lys)** | 10 | 0 | 6 | 2 | 3 | 6 | 4 | 4 | 7 | 2 | 7 | 1 | 52 | **100** |
| **TTG (Leu)** | 7 | 0 | 7 | 3 | 13 | 4 | 4 | 1 | 9 | 3 | 7 | 5 | 63 | 11.62 |
| **TTA (Leu)** | 30 | 7 | 34 | 19 | 40 | 46 | 12 | 10 | 29 | 13 | 60 | 22 | 322 | **59.41** |
| **CTG (Leu)** | 1 | 1 | 0 | 0 | 0 | 3 | 0 | 1 | 0 | 0 | 1 | 0 | 7 | 1.29 |
| **CTA (Leu)** | 6 | 2 | 14 | 4 | 2 | 10 | 5 | 3 | 4 | 1 | 8 | 2 | 61 | 11.25 |
| **CTT (Leu)** | 13 | 3 | 13 | 7 | 5 | 7 | 5 | 4 | 9 | 2 | 10 | 6 | 84 | 15.5 |
| **CTC (Leu)** | 0 | 0 | 1 | 0 | 1 | 0 | 1 | 1 | 0 | 0 | 0 | 1 | 5 | 0.92 |
| **ATG (Met)*** | 8 | 2 | 5 | 2 | 0 | 19 | 5 | 3 | 3 | 3 | 9 | 4 | 63 | **100** |
| **AAA (Asn)** | 8 | 1 | 13 | 3 | 15 | 1 | 3 | 7 | 9 | 5 | 11 | 3 | 79 | 37.09 |
| **AAT (Asn)** | 10 | 4 | 10 | 5 | 14 | 20 | 8 | 3 | 8 | 4 | 26 | 6 | 118 | **55.4** |
| **AAC (Asn)** | 0 | 1 | 2 | 2 | 1 | 2 | 1 | 2 | 0 | 1 | 3 | 1 | 16 | 7.51 |
| **CCG (Pro)** | 1 | 0 | 0 | 0 | 1 | 4 | 0 | 0 | 0 | 0 | 0 | 0 | 6 | 7.5 |
| **CCA (Pro)** | 2 | 0 | 1 | 2 | 0 | 4 | 1 | 0 | 0 | 0 | 1 | 1 | 12 | 15 |
| **CCT (Pro)** | 8 | 0 | 6 | 5 | 1 | 13 | 4 | 1 | 4 | 2 | 5 | 2 | 51 | **63.75** |
| **CCC (Pro)** | 0 | 0 | 4 | 0 | 0 | 2 | 1 | 1 | 1 | 0 | 2 | 0 | 11 | 13.75 |
| **CAG (Gln)** | 2 | 0 | 0 | 0 | 0 | 1 | 0 | 0 | 0 | 0 | 2 | 0 | 5 | 14.29 |
| **CAA (Gln)** | 4 | 0 | 0 | 3 | 2 | 6 | 6 | 1 | 3 | 1 | 2 | 2 | 30 | **85.71** |
| **CGG (Arg)** | 0 | 0 | 0 | 1 | 0 | 0 | 0 | 0 | 0 | 0 | 1 | 0 | 2 | 5.41 |
| **CGA (Arg)** | 2 | 1 | 2 | 1 | 1 | 3 | 1 | 0 | 2 | 0 | 2 | 0 | 15 | 40.54 |
| **CGT (Arg)** | 1 | 0 | 3 | 1 | 0 | 3 | 2 | 2 | 5 | 0 | 2 | 0 | 19 | **54.29** |
| **CGC (Arg)** | 1 | 0 | 0 | 0 | 0 | 0 | 0 | 0 | 0 | 0 | 0 | 0 | 1 | 2.7 |
| **AGG (Ser)** | 4 | 3 | 9 | 4 | 6 | 4 | 0 | 1 | 2 | 1 | 6 | 2 | 42 | 12.21 |
| **AGA (Ser)** | 6 | 4 | 11 | 3 | 6 | 7 | 4 | 5 | 8 | 2 | 11 | 4 | 71 | 20.64 |
| **AGT (Ser)** | 4 | 1 | 8 | 2 | 1 | 11 | 3 | 2 | 5 | 1 | 8 | 2 | 48 | 13.95 |
| **AGC (Ser)** | 1 | 0 | 5 | 0 | 2 | 2 | 3 | 2 | 1 | 1 | 4 | 5 | 26 | 7.56 |
| **TCG (Ser)** | 1 | 0 | 1 | 0 | 0 | 0 | 0 | 0 | 0 | 1 | 0 | 1 | 4 | 1.16 |
| **TCA (Ser)** | 2 | 1 | 2 | 0 | 1 | 6 | 2 | 0 | 2 | 0 | 4 | 1 | 21 | 6.1 |
| **TCT (Ser)** | 11 | 3 | 12 | 4 | 11 | 16 | 6 | 6 | 8 | 4 | 21 | 6 | 118 | **34.3** |
| **TCC (Ser)** | 1 | 0 | 4 | 1 | 3 | 0 | 2 | 0 | 0 | 0 | 2 | 1 | 14 | 4.07 |
| **ACG (Thr)** | 0 | 1 | 1 | 2 | 0 | 0 | 0 | 0 | 1 | 0 | 4 | 0 | 9 | 8.18 |
| **ACA (Thr)** | 5 | 0 | 3 | 2 | 0 | 5 | 1 | 1 | 2 | 1 | 2 | 1 | 23 | 20.91 |
| **ACT (Thr)** | 7 | 2 | 10 | 4 | 3 | 12 | 3 | 2 | 7 | 1 | 9 | 7 | 67 | **60.91** |
| **ACC (Thr)** | 1 | 1 | 0 | 0 | 2 | 2 | 1 | 1 | 1 | 0 | 0 | 2 | 11 | 10 |
| **GTG (Val)** | 6 | 0 | 1 | 2 | 0 | 5 | 2 | 1 | 1 | 1 | 5 | 1 | 25 | 10.96 |
| **GTA (Val)** | 7 | 2 | 6 | 4 | 2 | 17 | 6 | 5 | 3 | 3 | 7 | 5 | 67 | 29.39 |
| **GTT (Val)** | 22 | 1 | 7 | 5 | 14 | 20 | 10 | 2 | 10 | 8 | 17 | 9 | 125 | **54.82** |
| **GTC (Val)** | 1 | 0 | 1 | 1 | 2 | 2 | 1 | 0 | 1 | 1 | 1 | 0 | 11 | 4.82 |
| **TGG (Trp)** | 5 | 0 | 4 | 1 | 2 | 2 | 2 | 0 | 1 | 1 | 1 | 1 | 20 | 29.85 |
| **TGA (Trp)** | 5 | 1 | 4 | 2 | 0 | 14 | 2 | 4 | 2 | 1 | 6 | 6 | 47 | **70.15** |
| **TAT (Tyr)** | 23 | 0 | 22 | 3 | 22 | 17 | 12 | 11 | 17 | 5 | 38 | 17 | 183 | **87.98** |
| **TAC (Tyr)** | 2 | 2 | 0 | 0 | 1 | 6 | 0 | 3 | 3 | 2 | 3 | 3 | 25 | 12.02 |
| **TAG (Stop)** | 1 | 0 | 1 | 0 | 1 | 1 | 1 | 0 | 1 | 0 | 0 | 0 | 6 | 50 |
| **TAA (Stop)** | 0 | 1 | 0 | 1 | 0 | 0 | 0 | 1 | 0 | 1 | 1 | 1 | 6 | 50 |

* canonical start-codon for flatworm mitochondrial DNA; ** alternative start-codon suggested for ND2 and ND4
