# Supplementary material for: The mitochondrial genome of the egg-laying flatworm Aglaiogyrodactylus forficulatus (Platyhelminthes: Monogenoidea)
Source: Parasit Vectors. 2016 May 17;9:285. doi: 10.1186/s13071-016-1586-2 (PMC4869361; doi:10.1186/s13071-016-1586-2)
Supplement: Additional file 3: Figure S1. — The secondary structures of the tRNAs. (PDF 184 kb) [file 13071_2016_1586_MOESM3_ESM.pdf]

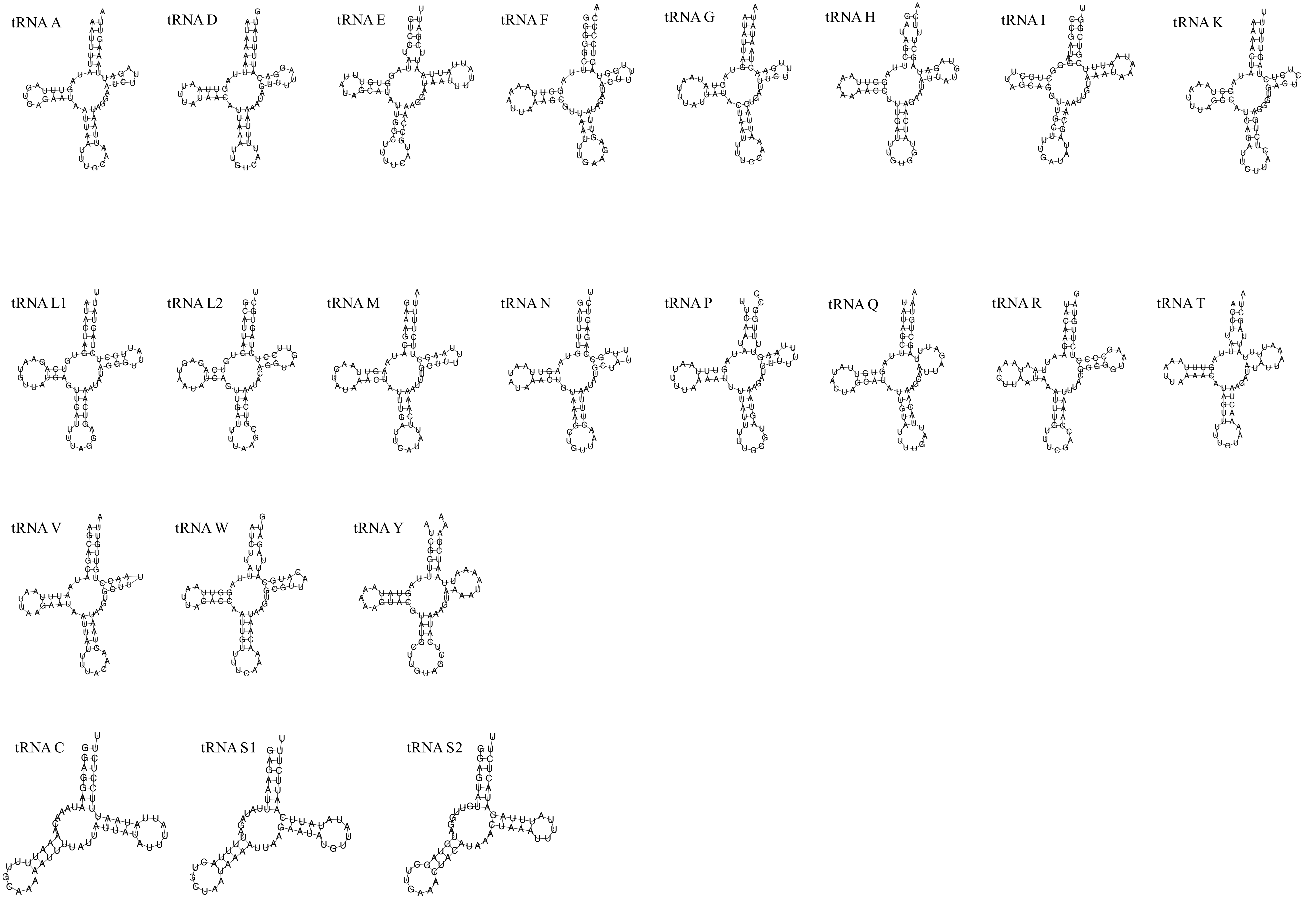

Additional File 3: The secondary cloverleaf structures of the 22 mitochondrial tRNAs of *A. forficulatus* as predicted by DOGMA [9]. Note that tRNAs C, S1, and S2 lack the DHU arm.
