# Supplementary material for: The mitochondrial genome of the egg-laying flatworm Aglaiogyrodactylus forficulatus (Platyhelminthes: Monogenoidea)
Source: Parasit Vectors. 2016 May 17;9:285. doi: 10.1186/s13071-016-1586-2 (PMC4869361; doi:10.1186/s13071-016-1586-2)
Supplement: Additional file 4: Figure S2. — Maximum Likelihood trees after removal of ambiguous sites. (PDF 119 kb) [file 13071_2016_1586_MOESM4_ESM.pdf]

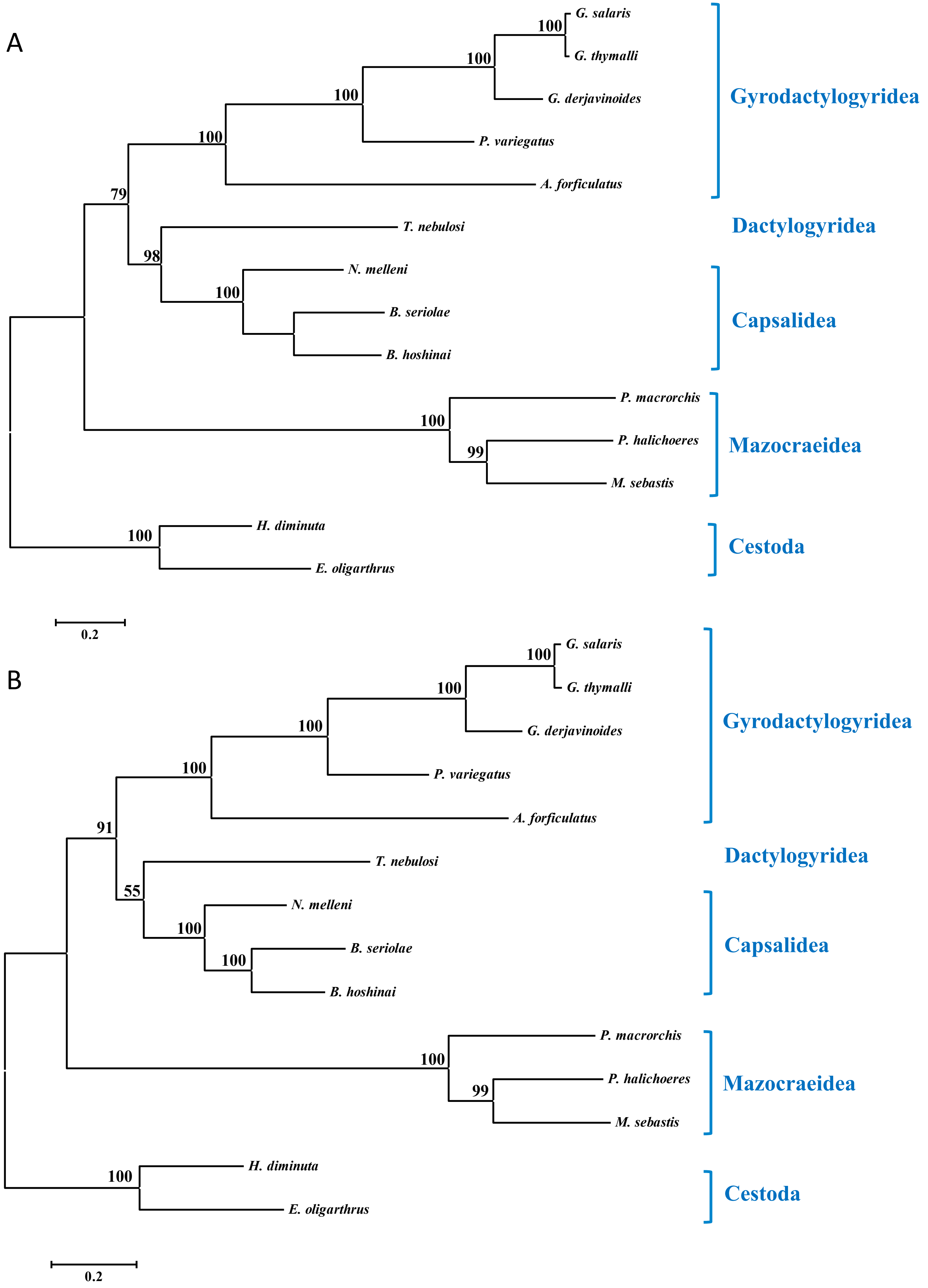

Additional File 4: Maximum Likelihood tree based on the concatenated MAFFT alignments of mitochondrial genes of *A. forficulatus* and 11 further Monogenoidea species after removal of ambiguous sites by (A) GBlock v0.91b [28] and (B) GUIDANCE2 [29]. The cestode species *H. diminuta* and *E. oligarthrus* served as outgroup. Bootstrap support values are indicated.
