# Supplementary material for: The mitochondrial genome of the egg-laying flatworm Aglaiogyrodactylus forficulatus (Platyhelminthes: Monogenoidea)
Source: Parasit Vectors. 2016 May 17;9:285. doi: 10.1186/s13071-016-1586-2 (PMC4869361; doi:10.1186/s13071-016-1586-2)
Supplement: Additional file 5: Table S3. — P-distances of concatenated mitochondrial genes. (DOCX 17 kb) [file 13071_2016_1586_MOESM5_ESM.docx]

**Additional File 5:** P-distances of pairwise comparisons of all concatenated mitochondrial genes of the complete MAFFT alignment (below diagonal, black font), and after removal of ambiguous sites by GBlock (above diagonal, blue font). The values for *Gyrodactylus salaris*, *G. thymalli*, and *G. derjavinoides* were averaged.

|  | *A. forficulatus* | *P. variegatus* | *G. spp.* | *B. seriolae* | *B. hoshinai* | *N. melleni* | *T. nebulosi* | *P. halichoeres* | *M. sebastis* | *P. macrorchis* |
| --- | --- | --- | --- | --- | --- | --- | --- | --- | --- | --- |
| *A. forficulatus* |  | 0.396 | 0.448 | 0.408 | 0.411 | 0.402 | 0.443 | 0.464 | 0.471 | 0.468 |
| *P. variegatus* | 0.402 |  | 0.340 | 0.397 | 0.398 | 0.386 | 0.428 | 0.450 | 0.455 | 0.462 |
| *G. spp.* | 0.456 | 0.357 |  | 0.449 | 0.450 | 0.442 | 0.456 | 0.497 | 0.502 | 0.496 |
| *B. seriolae* | 0.415 | 0.404 | 0.459 |  | 0.245 | 0.276 | 0.385 | 0.448 | 0.452 | 0.456 |
| *B. hoshinai* | 0.416 | 0.406 | 0.251 | 0.251 |  | 0.280 | 0.382 | 0.438 | 0.441 | 0.455 |
| *N. melleni* | 0.409 | 0.392 | 0.450 | 0.286 | 0.290 |  | 0.389 | 0.452 | 0.453 | 0.447 |
| *T. nebulosi* | 0.448 | 0.436 | 0.394 | 0.394 | 0.397 | 0.389 |  | 0.459 | 0.462 | 0.473 |
| *P. halichoeres* | 0.471 | 0.457 | 0.505 | 0.457 | 0.458 | 0.447 | 0.466 |  | 0.293 | 0.336 |
| *M. sebastis* | 0.477 | 0.463 | 0.509 | 0.460 | 0.461 | 0.450 | 0.471 | 0.308 |  | 0.338 |
| *P. macrorchis* | 0.474 | 0.469 | 0.503 | 0.464 | 0.462 | 0.455 | 0.480 | 0.351 | 0.354 |  |
