# Supplementary material for: The mitochondrial genome of the egg-laying flatworm Aglaiogyrodactylus forficulatus (Platyhelminthes: Monogenoidea)
Source: Parasit Vectors. 2016 May 17;9:285. doi: 10.1186/s13071-016-1586-2 (PMC4869361; doi:10.1186/s13071-016-1586-2)
Supplement: Additional file 6: Figure S3. — Schematic order of the mitochondrial genes. (PDF 113 kb) [file 13071_2016_1586_MOESM6_ESM.pdf]

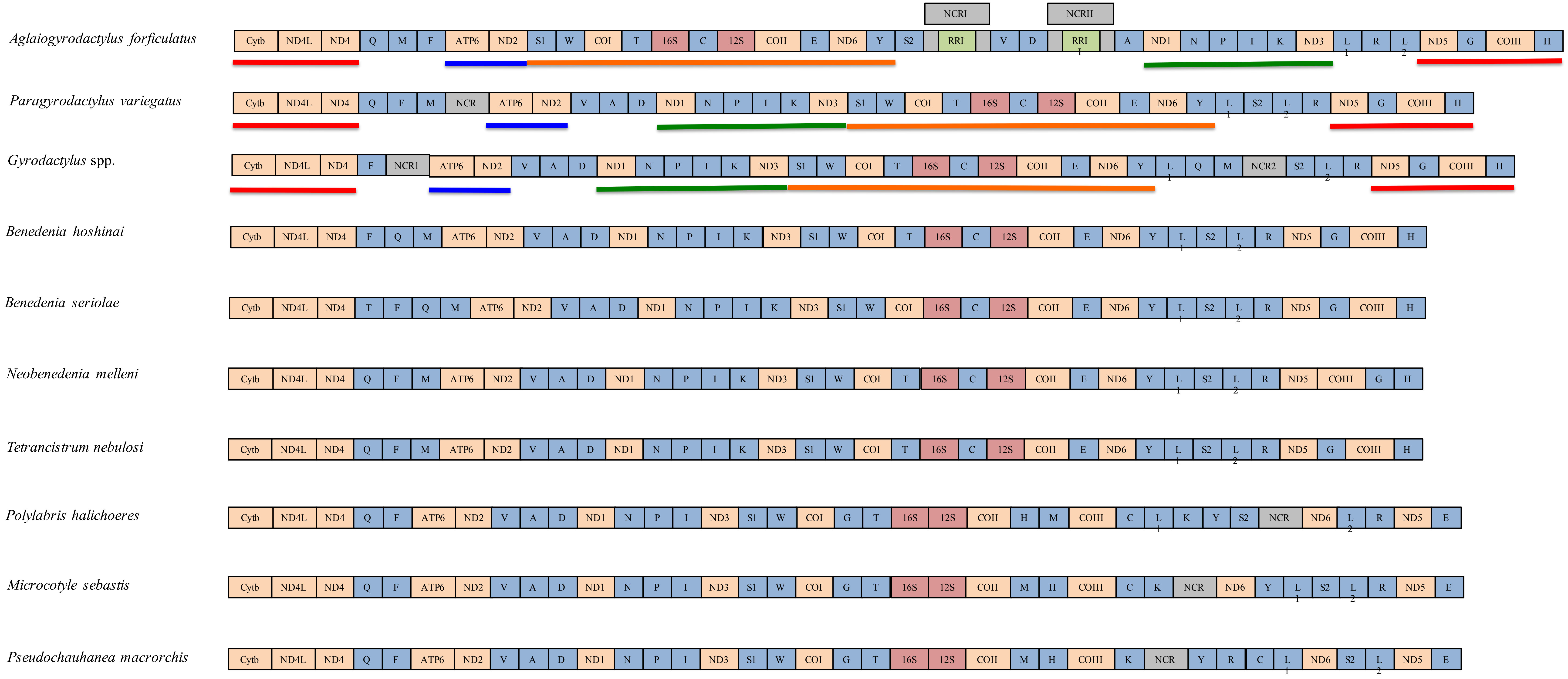

Additional File 6: Schematic order of the mitochondrial genes of *A. forficulatus* and 11 further monogenoid species. *Gyrodactylus salaris*, *G. thymalli*, and *G. derjavinoides* share the same gene order and are therefore collapsed into *Gyrodactylus* spp. The four conserved regions discussed in the text are depicted by different colors below the gene order sketches of the Gyrodactylidae species, i.e. red, blue, orange, and green. Box sizes do not correlate to gene lengths.
