# Supplementary material for: The mitochondrial genome of the egg-laying flatworm Aglaiogyrodactylus forficulatus (Platyhelminthes: Monogenoidea)
Source: Parasit Vectors. 2016 May 17;9:285. doi: 10.1186/s13071-016-1586-2 (PMC4869361; doi:10.1186/s13071-016-1586-2)
Supplement: Additional file 7: Figure S4. — Nucleotide sequence alignment of the repeat regions I and II. (DOCX 82 kb) [file 13071_2016_1586_MOESM7_ESM.docx]

**Repeat region I (positions 9017 – 9277)**

Repeat1 TTTTTTTAGA TTTGTT-ATA –AAAATTTTC AGGTACTATC AGCCTAAAAA

Repeat2 .......C.T ...T..TC.. TTTTT....T .....T.T.A .AG.......

Repeat3 .......C.T ...T..TC.. TTTTT..... .......... ..........

Repeat1 AAAAACGCTA AATTTGCTAT GAGGAAGTAA GCACAAAA

Repeat2 .......... .......... .......... ........

Repeat3 .......... ......G.G. .-..T.A.T. .T...T.G

**Repeat region II (positions 9539 – 10212)**

Repeat1 ATATTTTATT –ATGTAATAA TTATATTTTT TTATTTAAAA AATTTTATGC

Repeat2 .......C.. T......... .......... .......... ..........

Repeat3 .......C.. T......... .......... .......... ..........

Repeat1 TTTATTTAAG CCTAAAATTA GATAGTAAAA TTTTTATTTT TTTAAAATAA

Repeat2 .......... .......... .......... .......... ..........

Repeat3 .......... .......... .......... .......... ..........

Repeat1 AAATTAGGCT GTAATGAGCT TGTTTCACAA AAATACCTAG ATATATTAAA

Repeat2 .......... .......... .......... .......... ..........

Repeat3 .......... .......... .......... .......... ..........

Repeat1 GACGTTAGTC TTTGTATTTT GGTTTATTTT TTTAAGCTTA GTTTAATTAG

Repeat2 .......... .......... .......... .......... ..........

Repeat3 .......... .......... .......... .....T.C.. .C........

Repeat1 TGTGTTTATT AGATAACTAT ATTTTTAATA ATT

Repeat2 .......... .......... .......... ...

Repeat3 .C.A..G..

**Additional File 7:** Nucleotide sequence alignment of the repeat regions I (88 bp repeats) and II (233 bp repeats) in the respective non-coding regions (NCRs) I and II of the mitochondrial genome of *A. forficulatus* as identified by YASS [12].
