# Supplementary material for: The mitochondrial genome of the egg-laying flatworm Aglaiogyrodactylus forficulatus (Platyhelminthes: Monogenoidea)
Source: Parasit Vectors. 2016 May 17;9:285. doi: 10.1186/s13071-016-1586-2 (PMC4869361; doi:10.1186/s13071-016-1586-2)
Supplement: Additional file 8: Table S4. — CREx distance matrix for mitochondrial gene order rearrangements. (DOCX 16 kb) [file 13071_2016_1586_MOESM8_ESM.docx]

**Additional File 8:** CREx [32] distance matrix for the rearrangements of mitochondrial gene orders of *A. forficulatus* and 11 further monogenoid species. *Gyrodactylus salaris*, *G. thymalli*, and *G. derjavinoides* share the same gene order and are therefore collapsed into *Gyrodactylus* spp. The highest values for comparisons including *A. forficulatus* are highlighted.

|  | *P. variegatus* | *G. spp.* | *B. seriolae* | *B. hoshinai* | *N. melleni* | *T. nebulosi* | *P. halichoeres* | *M. sebastis* | *P. macrorchis* |
| --- | --- | --- | --- | --- | --- | --- | --- | --- | --- |
| *A. forficulatus* | **316** | 246 | 186 | 296 | 296 | **316** | 78 | 76 | 72 |
| *P. variegatus* |  | 638 | 576 | 1186 | 1186 | 1254 | 130 | 168 | 126 |
| *G. spp.* |  |  | 354 | 660 | 622 | 638 | 136 | 148 | 132 |
| *B. seriolae* |  |  |  | 608 | 544 | 576 | 116 | 154 | 112 |
| *B. hoshinai* |  |  |  |  | 1120 | 1186 | 124 | 162 | 120 |
| *N. melleni* |  |  |  |  |  | 1186 | 128 | 166 | 124 |
| *T. nebulosi* |  |  |  |  |  |  | 130 | 168 | 126 |
| *P. halichoeres* |  |  |  |  |  |  |  | 988 | 816 |
| *M. sebastis* |  |  |  |  |  |  |  |  | 810 |
